# Independent risk factors and the long-term outcomes for postoperative continuous renal replacement treatment in patients who underwent emergency surgery for type a acute aortic dissection

**DOI:** 10.1186/s13019-020-01153-8

**Published:** 2020-05-15

**Authors:** Zhigang Wang, Min Ge, Tao Chen, Cheng Chen, Qiuyan Zong, Lichong Lu, Dongjin Wang

**Affiliations:** Department of Cardiothoracic Surgery, Nanjing Drum Tower Hospital, The Affiliated Hospital of Nanjing University Medical School, Nanjing, 210008 China

**Keywords:** Aortic dissection, Continuous renal replacement therapy, Outcomes, Serum creatinine, Cardiopulmonary bypass, Thoracic aortic surgery

## Abstract

**Objective:**

The study objective was to investigate the incidence and risk factors of continuous renal replacement therapy (CRRT) in patients undergoing emergency surgery for type A acute aortic dissection (TA-AAD) and evaluate the perioperative and long-term outcomes.

**Methods:**

From January 2014 to December 2018, 712 consecutive patients were enrolled in the study. These patients were divided into two groups according to whether or not needed postoperative CRRT: the CRRT group vs the control group. Univariate analysis and binary logistic regression analysis were used to analyze the risk factors of CRRT. To avoid the selection bias and confounders, baseline characteristics were matched for propensity scores. Kaplan-Meier curves were generated to provide survival estimates at postoperative points in time.

**Results:**

Before propensity score matching, univariate analysis showed that there were significant differences in age, preoperative hypertension, pericardial effusion, preoperative serum creatinine (sCr), intraoperative need for combined coronary artery bypass grafting (CABG) or mitral valve or tricuspid valve surgery, cardiopulmonary bypass (CPB) time, extracorporeal circulation assistant time, aortic cross-clamp time, drainage volume 24 h after surgery and ventilator time between two groups. All were higher in the CRRT group (*P* < 0.05). These risk factors were included in binary logistic regression. It showed that preoperative sCr and CPB time were independent risk factors for CRRT patients undergoing surgery for TA-AAD. And there were significant differences regarding 30-day mortality (*P* < 0.001) and long-term overall cumulative survival (*P* < 0.001) with up to a 6-year follow-up. After propensity scoring, 29 pairs (58 patients) were successfully matched. Among these patients, the analysis showed that CPB time was still significantly longer in the CRRT group (*P* = 0.004), and the 30-day mortality rate was also higher in this group (44.8% vs 10.3%; *P* = 0.003).

**Conclusion:**

CRRT after TA-AAD is common and worsened short- and long- term mortality. The preoperative sCr and CPB time are independent risk factors for postoperative CRRT patients. Shorten the CPB time as much as possible is recommended to reduce the risk of CRRT after the operation.

## Introduction

Previous studies have shown a high occurrence of acute kidney injury (AKI) in patients undergoing cardiothoracic surgery procedures. It is related to increased mortality, morbidity, longer intensive care unit (ICU) stay time and reduces late survival [1–4]. Continuous renal replacement therapy (CRRT) is an effective treatment strategy for those patients with acute renal failure (ARF), especially those who require both circulatory and respiratory support.

Although mild to moderate acute kidney injuries has a high incidence [5], 2–15% of patients with AKI need CRRT after aortic surgery, and especially higher in those with TA-AAD surgery [1, 6, 7]. Despite continued progress in renal replacement therapy and intensive care in the past years, the short- and long-term mortality still remains high, with a rate ranging from 50% to over 80% in patients undergoing CRRT [8]. However, there is scarce literature on the incidence, risk factors and long-term outcomes for CRRT in patients with TA-AAD repair. Identification of risk factors for CRRT in patients with TA-AAD may lead to timely initiation of CRRT, and improve clinical outcomes. This retrospective study aims to identify the risk factors and 30-day and long-term outcomes for CRRT after surgical repair in patients with TA-AAD.

## Methods and materials

A total of 730 consecutive patients with TA-AAD in our hospital from January 2014 to December 2018 were retrospectively analyzed. Previous approval was obtained from the institutional research ethics committee, which waived the need for individual informed consent. Aortic dissection was diagnosed by computed tomography angiography at either our institution or the referring hospital. Patients with a history of chronic renal failure (CRF) or with intraoperative and postoperative death within 24 h were excluded. Patients were divided into the control group (601 cases) and the CRRT group (111 cases) according to whether they received CRRT after surgery. The basic clinical data of the two groups are shown in Table 1.

**Table 1:** Baseline clinical characteristics of patient population and aortic dissection features before and after propensity score matching

Characteristics	Before Matching			After Matching		
	Control(*n* = 601)	CRRT(*n* = 111)	*P* Value	Control(*n* = 29)	CRRT(n = 29)	*P* Value
Patient characteristics						
Demographics						
Age (year)	52.0 ± 13.0	55.4 ± 14.0	**0.011**	54.6 ± 13.2	54.8 ± 13.2	0.937
Male (%)	438(72.9)	84(75.7)	0.540	20(69.0)	23(79.3)	0.368
BMI (kg/m2)	25.2 ± 5.0	26.1 ± 5.0	0.146	25.8 ± 3.4	27.0 ± 5.0	0.278
Medical history						
Hypertension (%)	401(66.7)	91(82.0)	**0.001**	20(69.0)	21(72.4)	0.773
Diabetes mellitus (%)	11(1.8)	1(0.9)	0.703	0	0	–
Previous cardiac surgery (%)	28(4.7)	8(7.2)	0.260	0	0	–
Previous Coronary artery disease (%)	18(3.0)	9(8.1)	**0.025**	1(3.4)	2(6.9)	1.000
Cerebrovascular disease (%)	20(3.3)	4(3.6)	0.779	2(6.9)	1(3.4)	1.000
Pericardial effusion (%)	15(2.5)	13(11.7)	**< 0.001**	1(3.4)	1(3.4)	1.000
Preoperative laboratory data						
BUN (mmol/L)	7.0 ± 3.8	11.5 ± 8.2	**< 0.001**	7.8 ± 3.0	6.6 ± 1.9	0.063
sCr (μmol/L)	91.0 ± 48.4	176.0 ± 156.5	**< 0.001**	96.6 ± 46.7	90.1 ± 35.1	0.552
Aortic dissection features						
Blood supply of left renal artery (%)						
True lumen	326(64.2)	38(62.3)		14(58.3)	6(37.5)	
False lumen	133(26.2)	17(27.9)		9(37.5)	7(43.8)	
True lumen and false lumen	49(9.6)	6(9.8)	0.978	1(4.2)	3(18.8)	0.272
Blood supply of right renal artery (%)						
True lumen	390(76.8)	41(67.2)		18(75.0)	11(68.8)	
False lumen	91(17.9)	13(21.3)		4(16.7)	2(12.5)	
True lumen and false lumen	27(5.3)	7(11.5)	0.106	2(8.3)	3(18.8)	0.766

Data presented as n (%); mean ± standard deviation

*Abbreviations*: *BMI* body mass index, *BUN* blood urea nitrogen, *sCr* serum creatinine

Demographic variables included age, gender, body mass index (BMI), previous medical history (hypertension, diabetes, cardiac surgery, coronary artery disease, cerebrovascular disease), aortic dissection features (blood supply of renal artery) and pericardial effusion. Operation-related variables were the duration of cardiopulmonary bypass (CPB) and aortic cross-clamping, extracorporeal circulation assist, the duration of deep hypothermic circulatory arrest (DHCA). Laboratory variables included preoperative serum creatinine (sCr) and serum blood urea nitrogen (BUN) levels. Postoperative variables included drainage volume 24 h after surgery, duration of mechanical ventilation, ICU and hospital stay, and 30-day mortality.

Criteria for the initiation and termination of CRRT after severe AKI is referred to the guidelines for the clinical practice of AKI from the global organization for the improvement of renal prognosis: Kidney Disease Improving Global Outcomes (KDIGO) [9]. CRRT was considered in patients with the increase of sCr more than 26.5umol/L within 48 h after surgery or the urine volume was less than 0.5 ml/kg/h lasting for 6 h, and serum K^+^ > 6.0 mmol/L or HCO_3_^−^ < 10 mmol/L. Within 48 h after the last CRRT, if sCr decreased >50umol/L (the sampling interval greater than 12 h) or urine volume > 0.5 ml/kg/h within 12 h, serum K^+^ < 5.5 mmol/L and HCO_3_^−^ > 18 mmol/L, CRRT was considered for termination, as introduced in previous studies [10].

CRRT was performed in our department, using the 11.5f double-chamber dialysis catheter, the AV600S polysulfone membrane blood filter and the connection pipeline of blood filtration, infusion pump, and syringe pump. The internal jugular vein or femoral vein or subclavian vein was selected to place a single double-chamber blood filter catheter. The hemodynamic force is provided by the blood pump. 1000 ml heparin brine was pre-flushed before using the filter to empty the air bubbles in the filter and pipeline. We then placed the sterile collecting bag 30-50 cm below the filter, and recorded the flow of liquid in and out every hour. In the early postoperative patients after aortic dissection, local anticoagulation of prefilter citrate was used to reduce bleeding. The replacement solution was 0.9% normal saline and 5% glucose solution, with a ratio of 3:1. Additionally, 250 ml 5% sodium bicarbonate was added for q4h or q6h to timely supplement the physiological needs and nutrients lost by blood filtration. The input method is pre or post-dilution method, which can balance the fluid and adjust the infusion speed according to the amount of filtrate and input. The filter is usually replaced after a blockage or when the filtrate drops, and the continuous veno-venous hemofiltration (CVVH) blood flow should be 100-150 ml/min. To maintain perfusion pressure of kidney, vasopressor and inotropic drugs were routinely used in TA-AAD patients with postoperative hypotension.

### Surgical procedure

The median sternal incision was used in all surgeries under general anesthesia and DHCA. All patients were treated with Terumo inlet membrane lung, no pre-rinse containing sugar was used in extracorporeal circulation, ultrafiltration and autologous blood recovery devices were routinely used. Extracorporeal circulation was established by a routine femoral artery or right axillary artery and right atrial intubation. When the nasopharyngeal temperature dropped to 34 °C, the ascending aorta was clamped and cardiac arrest fluid was injected to complete the operation of the proximal end of the aorta. When the nasopharyngeal temperature dropped to 18 ~ 20 °C and the bladder temperature to 22 ~ 24 °C, systemic circulatory was arrested, and the flow was reduced to 3 ~ 5 ml· kg^− 1^·min^− 1^ to complete the operation of the aortic arch and descending arch. Near-infrared spectroscopy was used to monitor cerebral oxygenation in the bilateral frontal lobes during the DHCA time. All patients returned to the ICU for routine monitoring and treatment.

### Statistical analysis

SPSS 25.0 software was used for statistical analysis. Univariate analysis was performed for each variable. Data were compared using the Student *t* test or non- parametric Wilcoxon Mann-Whitney *U* test for continuous variables and the chi-squared or Fisher’s exact test for categorical variables before matching; The multivariate model included variables that were significant on univariate analysis. Baseline characteristics (Demographic variables, previous medical history, aortic dissection features and pericardial effusion) were matched for propensity scores. We performed one-to-one pair matching using nearest neighbor matching without replacement within 0.02 standard deviations of the logit of the propensity score as caliper width.

Kaplan-Meier curves were generated to provide survival estimates at postoperative points in time. Differences between the 2 groups were determined by log-rank tests. For all analyses, a probability value of less than 0.05 was considered statistically significant.

## Results

Three patients with CRF before surgery and 15 patients who died intraoperatively or within 24 h after surgery were excluded, a total of 712 patients were included in the study. Among them, 111 cases (15.9%) underwent CRRT after thoracic aortic surgery. For the CRRT group, there were 84 cases (75.7%) of males, the medical history included hypertension in 91 cases (82%), diabetes in 1 case (0.9%), coronary heart disease in 9 cases (8.1%), cardiovascular surgery in 8 cases (7.2%), cerebrovascular accident in 4 cases (3.6%), and pericardial effusion in 13 cases (11.7%). (Table 1) The operation methods included aortic root or ascending aorta replacement in 49 cases (44.1%), total aortic arch replacement in 62 cases (55.9%), and aortic valve treatment in 31 cases (27.9%), coronary artery bypass grafting (CABG) or mitral or tricuspid valve operation in 17 cases (15.3%). One hundred seven patients (96.4%) received emergency operations. The mean CPB duration was 273.4 ± 80.6 min, the duration of aortic cross-clamp was 186.0 ± 70.5 min, and the mean duration of DHCA was 30.2 ± 50.1 min, as shown in Table 2.

**Table 2:** Operative data and outcome before and after propensity score matching

Characteristics	Before Matching			After Matching		
	Control(*n* = 601)	CRRT(*n* = 111)	*P* Value	Control(*n* = 29)	CRRT(n = 29)	*P* Value
Operative procedures						
Total arch replacement (%)	309(51.4)	62(55.9)	0.389	16(55.2)	20(69.0)	0.279
CABG/MVR/MVP/TVP (%)	51(8.5)	17(15.3)	**0.024**	5(17.2)	4(13.8)	1.000
Aortic valve (%)	185(30.8)	31(27.9)	0.548	9(31.0)	7(24.1)	0.557
CPB-related profiles						
CPB time (min)	235.5 ± 64.1	273.4 ± 80.6	**< 0.001**	236.0 ± 48.8	279.3 ± 59.8	**0.004**
Extracorporeal circulation assist time (min)	54.3 ± 31.0	65.9 ± 40.4	**0.001**	50.3 ± 30.7	60.4 ± 24.5	0.174
Aortic cross-clamp time (min)	165.3 ± 55.1	186.0 ± 70.5	**0.005**	174.6 ± 48.2	191.8 ± 50.9	0.192
DHCA time (min)	28.0 ± 12.7	30.2 ± 50.1	0.117	26.8 ± 10.4	31.3 ± 13.9	0.173
Complications and short-time outcomes						
Drainage volume 24 h after surgery (ml)	695.0 ± 604.1	1091.0 ± 1013.9	**0.001**	971.6 ± 1104.1	1011.5 ± 681.9	0.873
Ventilation time (hour)	32.0 ± 50.1	56.8 ± 61.8	**0.009**	37.6 ± 41.6	79.9 ± 81.9	0.058
30-day mortality (%)	40(6.7)	50(45.0)	**< 0.001**	3(10.3)	13(44.8)	**0.003**
ICU Stay time (day)	6.5 ± 11.5	9.4 ± 6.6	**0.020**	5.9 ± 5.7	9.3 ± 6.9	0.055
Hospital stay time (day)	22.3 ± 11.6	22.5 ± 17.0	0.892	19.4 ± 10.0	21.3 ± 14.3	0.560

Data presented as n (%); mean ± standard deviation

*Abbreviations*: *CABG* coronary artery bypass graft, *MVR* mitral valve replacement, *MVP* mitral valvuloplasty, *TVP* tricuspid valvuloplasty, *CPB* cardiopulmonary bypass, *DHCA* deep hypothermic circulatory arrest, *ICU* intensive care unit

Compared with the CRRT group, preoperative age, hypertension, pericardial effusion, preoperative sCr and BUN, intraoperative need for combined CABG or mitral valve or tricuspid valve surgery, CPB time, extracorporeal circulation assistant time, aortic cross-clamp time, drainage volume 24 h after surgery, ventilator time, 30-day mortality, ICU time between two groups, all above results were higher in CRRT group (*P* < 0.05). There was no significant difference in hospital stay between the two groups. (Tables 1, 2).

Binary logistic regression analysis showed that preoperative sCr (*OR* = 1.008, 95%*CI*: 1.002–1.014, *P* = 0.005) and CPB time (*OR* = 1.022, 95%*CI*: 1.003–1.042, *P* = 0.026) were independent risk factors for CRRT after thoracic aortic surgery. (Table 3).

**Table 3:** Binary logistic regression analysis of Risk Factors for the groups with and without Dialysis

Variable	Odds Ratio	95% CI	*P* value
Age	1.052	0.984–1.052	0.312
Hypertension	2.027	0.645–6.376	0.227
Previous Coronary artery disease	0.319	0.021–4.866	0.412
Pericardial effusion	0.552	0.043–7.051	0.647
BUN	1.041	0.966–1.123	0.293
sCr	1.008	1.002–1.014	**0.005**
CABG/MVR/MVP/TVP	0.501	0.110–2.279	0.371
CPB time	1.022	1.003–1.042	**0.026**
Extracorporeal circulation assist time	0.997	0.980–1.014	0.721
Aortic cross-clamp time	0.987	0.967–1.008	0.229
Drainage volume 24 h after surgery	1.000	1.000–1.001	0.314
Ventilation time	1.006	0.998–1.014	0.131

*Abbreviations*: *CI* confidence interval, *BUN* blood urea nitrogen, *CABG* coronary artery bypass graft, *MVR* mitral valve replacement, *MVP* mitral valvuloplasty, *TVP* tricuspid valvuloplasty, *CPB* cardiopulmonary bypass

After propensity scoring, 29 pairs (58 patients) were successfully matched. In these patients, the analysis showed that CPB time was significantly longer in CRRT group (*P* = 0.004), and the 30-day mortality rate was also higher in this group (44.8% vs 10.3%; *P* = 0.003). (Table 2).

**Fig. 1:**
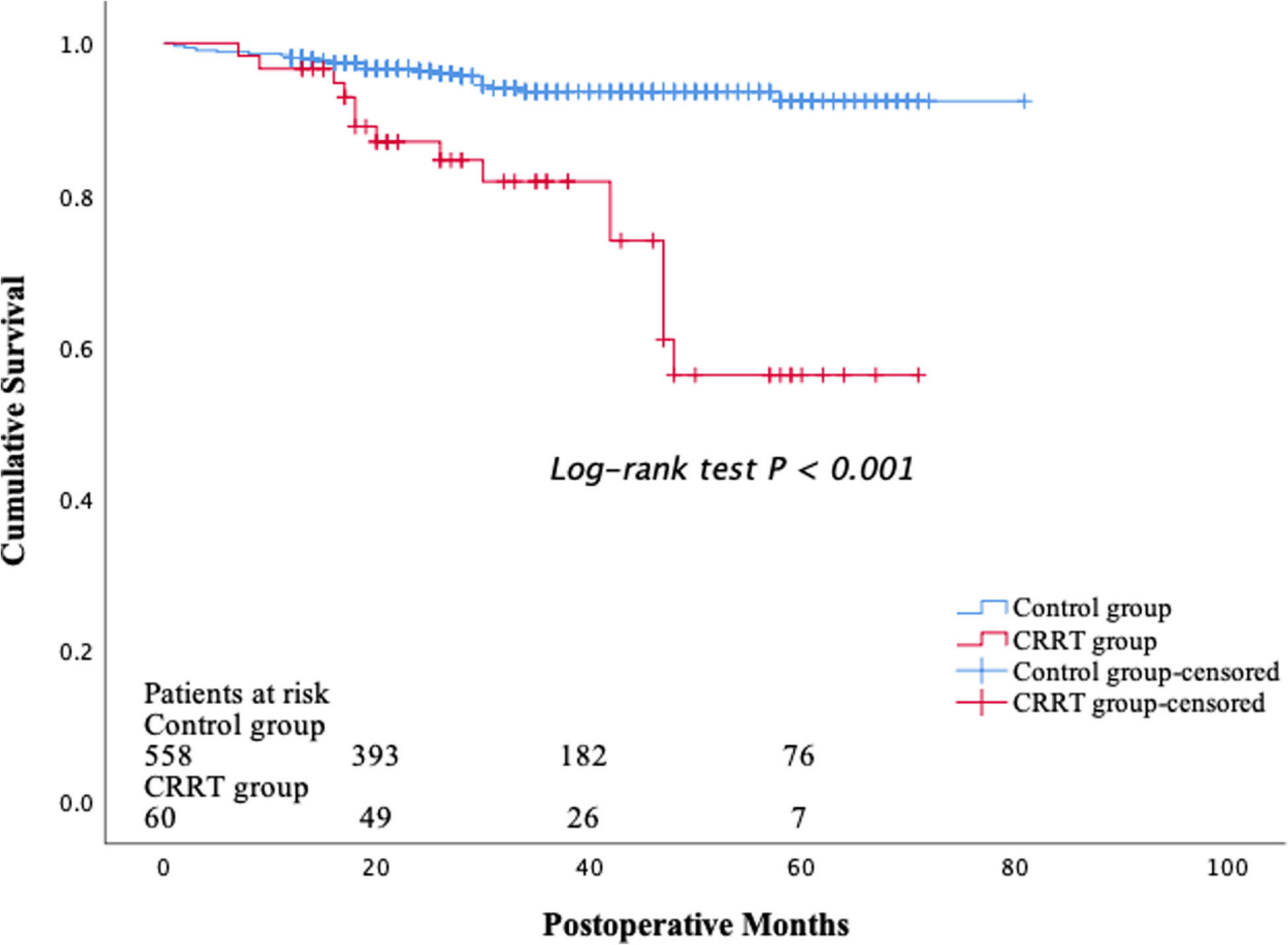
Long-term overall cumulative survival after surgical aortic repair for type A acute aortic dissection in CRRT and control groups with significant different risk profiles: Kaplan–Meier estimation. Patients were censored at the cut-off of the study

Fifty-one patients in the CRRT group and 43 patients in the control group died during the hospitalization period. At last, a total of 60 CRRT patients and 558 non-CRRT patients were included in the follow-up. The median follow-up time was 29 months. Forty-eight patients were lost from follow-up, including 47 patients in the control group and 1 in the CRRT group. They were excluded from the subsequent long-term survival analysis. There were 41 late deaths, it is important to point out that 1 patient in the control group committed suicide 6 months after discharge and was excluded from the study. Finally, 25 deaths (4.9%) in the control group and 15 (25.4%) in the CRRT group were identified (*P* < 0.001). The survival is shown in Fig. 1.

## Discussion

To the best of our knowledge, the study we report herein is the first one to investigate the risk factors and outcomes of emergent surgery for TA-AAD in postoperative CRRT patients. In this study, 111 patients (15.6%) suffered severe AKI after surgery and needed CRRT. The 30-day and long-term mortality rates were significantly higher in the CRRT group (*P* < 0.001). Our findings showed that in patients undergoing surgery for TA-AAD under the DHCA procedure, preoperative sCr and CPB time are independent risk factors for postoperative CRRT. To balance the selection bias and other confounders, baseline characteristics were matched for propensity scores. After matched, CPB time and 30-day mortality still showed significant differences between the two groups. Our Kaplan-Meier plots also revealed that the mortality of postoperative dialysis patients was significantly higher than that of the control group.

Consistent to our findings, Rice, R.D et al. [7] confirmed that the proportion of CRRT for such patients was 18.2%. In another study conducted by Estrera et al., 347 patients with typical type A aortic dissection were analyzed, 64 cases of which suffered from CRRT (18.4%) [6]. The incidence of postoperative CRRT in our cohort was comparable with these previous studies. CRRT procedure involved in the context of renal failure may lead to circulation instability, infection, thrombosis, and electrolyte imbalance, which will impair the postoperative recovery of patients, and even cause death [11], implying a low survival rate of the CRRT group as shown in our study. Elahi et al. suggested that early initiation of CRRT may reduce mortality and mortality in patients with severe AKI after cardiac surgery [12].

Elevated sCr levels before surgery was identified as a risk factor for CRRT in our study, which is consistent with previous reports [1, 13, 14]. Preoperative creatinine serum levels showed a significant influence on postoperative renal function. Elevated sCr levels represented damaged renal function. It is believed that increased (> 50%) sCr resulted from hypotension, nephrotoxins and inflammation may lead to renal function injury [15]. Hypotension may result from cardiac tamponade or dehydration in TA-AAD patients. Reduction of renal blood flow, whether generalized or localized, plays a critical role in the occurrence of AKI [16]. Nephrotoxins include drugs and contrasts, which directly make damage to the kidney. Inflammatory response plays a major role in the development of acute aortic dissection and may also contribute to the vascular and renal parenchyma stress response, leading to the occurrence of AKI. Therapeutic strategies aimed at reducing kidney damage and accelerating function recovery contain nonpharmacologic, pharmacologic, and dialytic approaches [17]. Therefore, any therapy that can treat hypotension, nephrotoxins and inflammation regarding the increase of sCr may be helpful to improve the outcome, such as fluid, colloid administration for hemodynamic optimization. We’d better pay more attention when dealing with such cases because we confirmed the relationship between elevated preoperative sCr level and postoperative CRRT in our study.

Besides the sCr level, the CPB time was identified another risk factor for CRRT, as reported by Wu and colleagues [14]. Renal hypotension and hypoperfusion during CPB may be the main causes [18]. With longer duration of CPB, more severe hypotensive periods may occur frequently. This entails prolonged period in which the mean arterial pressure is below the optimal renal autoregulation threshold [19], which may aggravate the inflammatory response and induce ischemic kidney damage [20]. Besides, circulation is stopped during aortic dissection surgery, and ischemia-reperfusion injury may occur during the operation, ischemia-reperfusion injury may lead to massive apoptosis of renal endothelial cells [21]. In addition, CPB procedure causes damage to red blood cells, and the resulting cell debris forms tiny emboli that block the renal tubular vascular network, resulting in a decrease in the filtration area of renal tubules [22, 23]. The released hemoglobin also acts as an endogenous toxin through the release of iron, especially in the presence of low ferritin level [24]. Whether patients recover without sequela or develop AKI by progressing into the “extension phase” of kidney injury largely depends on the severity of the ensuing inflammatory response, renal hypoxia, and oxidative stress [25]. All these factors become more important if CPB is prolonged. In patients at high risk of postoperative CRRT, reduced CPB duration should be considered in perioperative care.

Based on our findings, the mortality rate of the CRRT group is significantly higher than that of the control group on 30-day and long term postoperative outcomes. A report from Japan also found that in patients who underwent surgery for TA-AAD, the mortality and major adverse cardiovascular and cerebrovascular events were correlated significantly with the severity of AKI. Furthermore, multivariate analysis showed that AKI stage 3 was an independent risk factor for mortality in those patients [26]. Sasabuchi et al. [2] also reported that stage 3 AKI defined by the KDIGO criteria had a significantly lower survival rate after long-term follow-up, with a median survival time of 58 months. A meta-analysis of cohort studies found that patients with AKI had higher risk of experiencing chronic kidney disease and end-stage renal disease, and the risk increased with the severity of AKI [27]. Patients with chronic kidney disease are also reported to have an increased risk of cardiovascular mortality, which contributed to the poor prognosis [28]. In addition to blood pressure control and regular assessment of aortic size, which aims to prevent aortic rupture, education for smoking cessation and weight control, using medications to lower cholesterol and to control serum blood glucose level may improve long-term survival in these patients [29].

### Study limitations

Like any retrospective study of patients with TA-AAD, this study has some limitations. First of all, this is a single-center retrospective study, and there may be other confounding factors that influence the results. Secondly, despite propensity score matching was used, further potential bias and con-founders in the context of patient selection and treatment cannot be completely excluded, and the statistical power was limited due to a small patient cohort (29 pairs). Finally, our surgical technique has evolved over the study period, and our findings may have been influenced by the involvement of different surgeons.

## Conclusions

In conclusion, patients receiving CRRT treatment who underwent emergency surgery for TA-AAD had higher rates of perioperative mortality and postoperative morbidity. The preoperative serum creatinine and CPB time were independent risk factors for postoperative CRRT. Further prospective multicenter studies are needed to assess the prognostic significance of CRRT and establish the most effective strategies to prevent and treat postoperative CRRT and thereby improve patient outcomes.

## Data Availability

The datasets used or analyzed during the current study are available from the corresponding author on reasonable request.

## Abbreviations

CRRT: Continuous renal replacement therapy
TA-AAD: Type A acute aortic dissection
AKI: Acute kidney injury
sCr: Serum creatinine
CABG: Coronary artery bypass grafting
CPB: Cardiopulmonary bypass
ICU: Intensive care unit
ARF: Acute renal failure
CRF: Chronic renal failure
BMI: Body mass index
DHCA: Deep hypothermic circulatory arrest
BUN: Blood urea nitrogen
KDIGO: Kidney disease improving global outcomes
CVVH: Continuous veno-venous hemofiltration
MVR: Mitral valve replacement
MVP: Mitral valvuloplasty
TVP: Tricuspid valvuloplasty
CI: Confidence interval
